# The importance of long-term follow up of participants in clinical trials

**DOI:** 10.1038/s41416-022-02038-4

**Published:** 2022-12-01

**Authors:** Jack Cuzick

**Affiliations:** Wolfson Institute of Population Health, Queen Mary University of London, London, UK

**Keywords:** Medical research, Diseases

As good health is a lifetime issue, long-term follow up is an important part of evaluating any medical condition or treatment. This is well appreciated in epidemiologic studies where exposure to a harmful substance is often long-term, and its impact on health can appear many years after first occurrence. Examples include the classic study of Doll and Peto [1] on 34,439 male British doctors, which began in 1951 and last reported in 2004 after 50 years of follow up, in which lifelong cigarette smoking was shown to reduce life expectancy by an average of 10 years, but cessation at age 60, 50, 40 or 30 years reduced this by about 3, 6, 9, or almost the full 10 years, respectively. Other classic long-term epidemiologic studies have focussed on diet and alcohol consumption [2, 3], hormone replacement therapy [4], or have been more general and studied a wider set of risk factors, often among healthcare professionals; for example, the ACS Cancer Prevention Study [5], the Harvard-based Nurses Health Studies [6] and the Health Professionals Follow Up Study [7].

## Screening trials

Treatments for clinical problems are usually directed at an immediate health issue, and obtaining data on long-term sequelae may seem less relevant and can be expensive and difficult to organise, especially in countries which do not have national repositories containing the needed information. For these reasons, long-term follow up is often not pursued. Long-term consequences are much more relevant for screening interventions and prophylactic treatments aimed at preventing future cancer, such as the value of human papilloma virus (HPV) vaccination, or the importance of screening for breast and colorectal cancer. For example, for breast cancer screening with short term follow up many cancers were found which did not appear to require treatment, leading to the belief that up to 50% of the screen detected cancers were over-diagnosis of indolent cancers that would not be clinically detected in the woman’s lifetime [8], and this was a drawback of breast screening. However, with longer follow up [9] these cancers were found to be earlier diagnosis of clinically relevant disease, and it was estimated that of the total number of screen detected cancers in the screened population, only 9.5% overall were over-diagnosed, and this was reduced to 3.7% after adjustment for self-selection. Thus, long-term follow up, even after screening has ended, is necessary to accurately assess over-diagnosis. Also, additional procedures and late side-effects need to be recorded and analysed.

Early studies clearly demonstrated the value of the cytological Papanicolaou smear test in identifying cervical lesions at a treatable stage and reducing mortality. This has been well established for many decades, and the evidence was so clear that randomised clinical trials were not needed for confirmation [10], although several long-term cohort studies have demonstrated the benefits of screening in reducing cervix cancer mortality [11]. More recently, tests have been developed for HPV, which causes cervix cancer, and trials have been conducted in which all women receive both HPV and Pap tests, and those positive for either test are referred to colposcopy to directly compare the two tests in the same woman [12]. These trials have clearly shown that HPV tests detect more cervical lesions than cytology in a range of countries and settings. Four randomised European trials with extended individual patient follow up in 176,464 women have been conducted to compare the impact of an HPV test vs. the Pap test on subsequent cancer rates. After a 6.5-year median follow up, these trials have demonstrated that HPV testing reduces cancer incidence by a further 40% overall compared with cytology, and by 70% when the HPV test was negative [13]. Longer follow up of these studies is needed to demonstrate that this reduction in cancer incidence translates into a reduction in cervix cancer mortality. However, a large four-arm cluster randomised trial of 131,746 women in rural India has also reported substantial increases in detection of precursor lesions, and, after a 20-year follow up, a significant reduction in cervix cancer deaths (34 vs. 64), which was not seen with Pap cytology or visual inspection (VIA) was also demonstrated [14]. A range of trials have also been conducted that look at the duration of protection following a negative HPV test, and after a 6-year follow up, Dillner et al. [15] have demonstrated a roughly fourfold reduction in high-grade precursor lesions and cancer (CIN3+) after a negative HPV test compared with a negative cytology test. These findings have been confirmed in a recent systematic review [16]. Several studies have also shown that HPV testing can be done on a vaginal self-sample or a urine sample, and achieves similar sensitivity for high-grade CIN, although specificity was somewhat lower [17]. This approach promises to increase acceptance of cervical screening for women, especially for those who feel uncomfortable having their sample taken by a clinician. Additional follow up of these studies will be necessary to determine which self-sampling device is best and the most appropriate screening interval when self-sampling is performed.

The Flexi-Sig screening trial of once-in-a-lifetime sigmoidoscopy for preventing colorectal cancer randomised 170,034 individuals, aged 55–64 years, in a 2:1 ratio to a single sigmoidoscopy or no screening. After 17 years of follow up it has demonstrated that the higher detection of precursor lesions found with screening was followed by a 26% reduction overall in colorectal cancer incidence, and a 30% reduction in mortality, based on 41% and 46% reductions in distal cancer incidence and mortality respectively [18]. When restricted to those actually receiving screening, larger reductions were seen: 56% and 66%, respectively [18]. Similar results have been seen for the colorectal component of the PLCO trial [19] and elsewhere [20].

Prostate-specific antigen (PSA) screening for prostate cancer is a controversial subject, where even long-term follow up of a large number of trials has not resolved the major issues. While all investigators acknowledge that screening has led to a substantial amount of over-diagnosis and over-treatment, the extent of a reduction in prostate cancer mortality, if any, remains controversial. The large European ERSPC trial has reported a 20% reduction in mortality after a maximum of 16 years of follow up in 182,160 men [21] with increasing benefit occurring with longer follow up, and the number needed to treat to prevent one prostate cancer death falling from 48 after 9 years of follow up [22] to 27 after 13 years [23] and 18 after 16 years [21]. Support for this method of screening as also been expressed [24] from North American investigators [24]. However, other studies have not been so positive, with conclusions of little or no mortality benefit in large studies and concerns about side-effects associated with treatment of indolent disease. Notable among these are the PLCO trial, with 13-year follow up in 76,685 men [25], an individual patient overview of 5 trials of 727,718 men with a 10-year follow up [26], and a large overview view of 1,904,950 patients in 63 studies [27]. These studies compared an invitation for screening with usual care, and were conducted mostly in North America, for which there was likely to be a substantial amount of opportunistic screening in the control arm. These results indicate that study size and length of follow up are not the only relevant factors. Monitoring and follow up procedures also need to be examined. Selection of who needs screening, an efficient triage algorithm to determine who to biopsy following a positive screening test, and avoidance of over-treatment of likely indolent lesions are also areas that merit further research.

Major trials have also been conducted for ovarian cancer screening, with mixed results. The most recently reported was the UKCTOCS trial, which randomised women to an annual CA125 blood test (*N* = 50,640), annual ultrasound (*N* = 50,639), or no screening (*N* = 101,359). After a median follow up of 11.1 years, a much higher proportion of low-volume ovarian and peritoneal cancers was found in the CA125 group vs. controls (40% vs. 26%, *P* < 0.0001), along with a non-significant 16% lower ovarian cancer mortality (*P* = 0.23) [28], leading the investigators to continue blinded follow up for mortality. After a 16.3-year median follow up, the difference in stage distribution was maintained, but no relative improvement in ovarian cancer mortality was seen in either screened arm [29] (hazard ratio for CA125 group = 0.96 (0.83–1.10), *P* = 0.52), leading to the conclusion that the stage shift did not translate into a mortality gain.

Screening for lung cancer provides another example of the need to evaluate cause specific mortality and not just rely on the detection of better risk cancers. An early major trial of screening by chest X-ray and sputum cytology in 9211 male smokers was conducted by the Mayo Clinic from 1971–83, and early findings based on screen detected cancer indicated that they were more likely to be resectable, postsurgical Stage I or II, and associated with better 5-year survival [30]. However, after 20.5 years of follow up, non-significantly more lung cancer deaths were seen in the screened group (337 vs. 303, relative risk 1.13) [31], indicating a lead time bias in screen cases detected that did not translate into a mortality benefit. While chest X-ray was not effective, subsequent trials using more sensitive CT scans have been shown to reduce mortality [32–34] and are now widely used in smokers and other high-risk individuals.

## Prevention trials

**Fig. 1:**
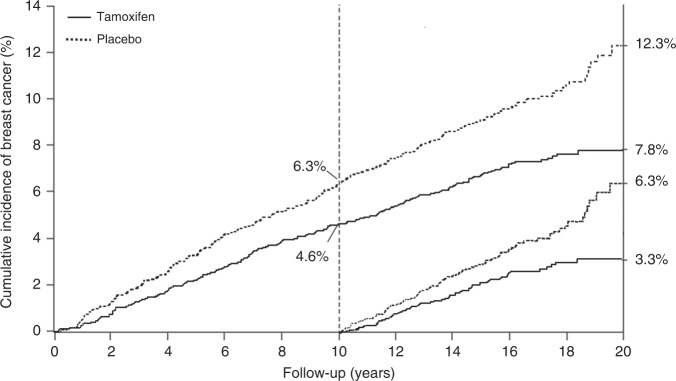
Impact of 5 years of tamoxifen on breast cancer incidence. 20-year incidence of breast cancer in high risk women receiving tamoxifen or placebo in the IBIS-I prevention trial. Incidence in the first 10 years and second 10 years of follow up is also shown separately.

Prevention trials are an example of research intermediate between epidemiologic studies and therapeutic treatment trials. Many of these studies have been done without randomisation, and in some cases this is appropriate. However, the most informative prevention trials have been randomised, and the extra information provided by this can be very valuable. As breast cancer is the commonest cancer in women in most countries, and is known to be related to hormone levels, it is an obvious candidate for preventive trials. The first major trials examined the role of tamoxifen as a preventive medicine. It was already accepted as an effective adjuvant treatment for reduction in progression of oestrogen receptor-positive breast cancer, and in those trials a reduction was seen in new contralateral tumours, which provided a strong basis for believing it could be effective for primary prevention. Four major trials have been undertaken: the largest was a North American trial known as P-1 [35], which recruited 13,388 high-risk women, but sadly was curtailed after 7 years median follow up, so no long-term information is available. An Italian trial in 5408 hysterectomised women [36], and the IBIS-I trial, which recruited 7154 women [37] and which was preceded by the Marsden ‘pilot study’ of 2494 high-risk women [38] have also been conducted. Follow up was 11 years in the Italian trial and 20 years in the Marsden trial. Long-term follow up is continuing in the IBIS-I trial, with a 16.0-year median follow up at last publication. Long-term follow up data in that trial proved very informative, and demonstrated that 5 years of tamoxifen in high-risk women without breast cancer could prevent almost 29% of cancers over a 20-year follow up period, and the preventive effect was virtually identical in the first and second 10-year follow up periods, suggesting that the preventive effect of 5 years of tamoxifen could last a lifetime (Fig. 1). The number needed to treat to prevent one cancer was reduced from 59 after 10 years of follow up to 22 after 20 years.

Trials of raloxifene, another selective oestrogen receptor modulator like tamoxifen, have also evaluated its effect on breast cancer occurrence. The initial MORE trial was aimed at reducing fracture rates in 7705 osteoporotic women, and after 4 years of treatment it was extended for another 4 years in the CORE trial, which combined the two active treatment arms into a simple active arm at the lower dose, and retained the placebo arm. After 8 years of follow up in the combined trials a large 66% reduction in new breast cancer was seen, with an even larger 76% reduction in oestrogen receptor-positive cancer [39]. This led to the STAR trial, which compared raloxifene with tamoxifen in women at high risk of breast cancer, and after an 81-month median follow up it was found to be 24% less effective than tamoxifen, but had fewer side-effects [40]. The reasons for its lower efficacy remain unclear, but it might be related to the fact that women in MORE/CORE were osteoporotic.

Two trials have looked at the role of aromatase inhibitors in breast cancer prevention, again supported by strong findings, both for preventing recurrence and new cancers in the adjuvant setting, where they were more effective than tamoxifen [41]. The North American trial known as MAP.3 [42] showed a very large 65% reduction in invasive cancers in the first 3 years of follow up, but unfortunately follow up was also curtailed after 35 months median follow up, before long-term data could be obtained. The IBIS-II trial comparing anastrozole with placebo in 3864 high-risk postmenopausal women has now reported, with a median follow up of 10.9 years [43]. It found that reductions on new cancers continued after the 5-year active treatment period, although they were non-significantly smaller than those seen during treatment (49% overall, 61% years 0–5, 36% subsequently). Further follow up is planned, and is necessary to see if a continued preventive effect will be seen in the extended long-term follow up period, as with tamoxifen. This clearly has a major impact on number needed to treat to prevent one cancer, and will be important for determining optimal use of aromatase inhibitors for prevention.

Vaccine trials provide another good example of the need for long-term follow up. Early trials showed clearly that vaccination against HPV is effective in preventing HPV infection [44, 45] and longer term follow up of these trials has now demonstrated that this protection against infection carries forward to reduce the incidence of precursor CIN lesions [46, 47], but further follow up is needed to see if the expected reduction in cervical cancer incidence and mortality can also be achieved.

**Fig. 2:**
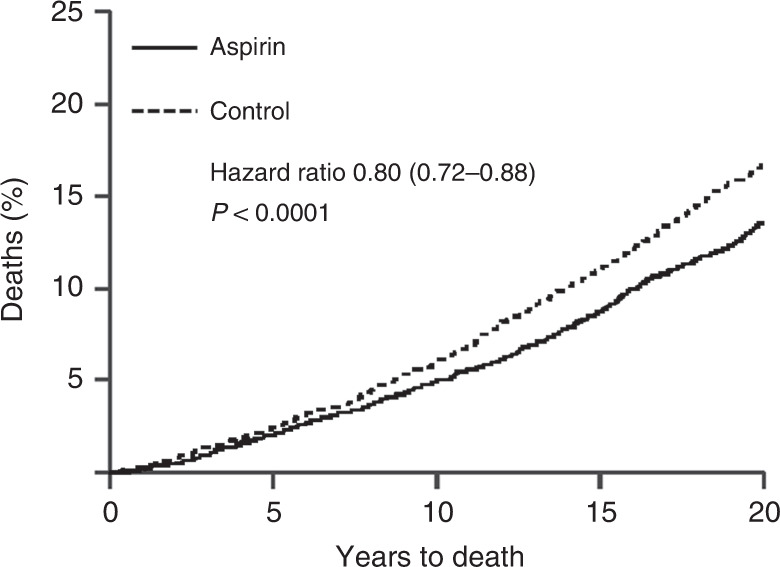
Twenty year impact of daily aspirin on deaths from all solid tumours (Rothwell et al. [48]).

Another somewhat serendipitous set of trials has also been conducted for low-dose aspirin. These trials, of at least 4 years of aspirin or control use, were originally designed to look at its impact on cardiovascular disease, and only short term follow up, typically for less than 5 years, was reported for this. However, Peter Rothwell and colleagues [48] resurrected these 8 trials, involving 25,570 patients, and obtained information on deaths and cancers for up to 20 years of follow up, which led to the discovery of important benefits for cancer prevention, primarily for colorectal, stomach and oesophageal cancers. Curiously, very little effect on cancer was seen in the first 5 years of follow up, but large effects were seen subsequently (Fig. 2), leading to an ~20% reduction in cancer deaths overall, a 34% reduction in the post 5-year-period for all cancer deaths, and a 54% reduction in gastrointestinal cancers, which was still diverging after 20 years of follow up. The role of long-term follow up was vital in discovering this pronounced preventive activity. The clearest evidence was for a reduction in colorectal cancer, but gastric and oesophageal cancer were also substantially reduced. These results have now been validated in several non-randomised epidemiologic studies [49, 50], and are summarised in Table 1 [51]. A current challenge for understanding the role of aspirin for cancer prevention comes from the results of two recent reports on studies with short follow up [52, 53]. Given what was known from cardiovascular studies, it should have been anticipated that no benefit would be seen in the first 5 years, and only the side-effects would be apparent. This is what has happened, and has been misinterpreted by many to conclude that aspirin has no effect on cancer when, in fact, no useful efficacy information has yet to be obtained from these studies [54].

**Table 1:** Estimated impact of daily aspirin on cancer, heart disease and gastrointestinal bleeding (Cuzick et al. [51]).

Event	Incidence	Mortality
	Relative risk	Relative risk
Colorectal cancer	0.65	0.60
Oesophageal cancer	0.70	0.50
Gastric cancer	0.70	0.65
Lung cancer	0.95	0.85
Prostate cancer	0.90	0.85
Breast cancer	0.90	0.95
Myocardial infarction	0.82	0.95
Stroke	0.95	1.21
Major bleeding	1.54	-
GI bleeding	-	1.60
Peptic ulcer	-	1.60

Negative effects are shown with a relative risk greater than unity.

The Prostate Cancer Prevention Trial (PCPT) compared the anti-androgen finasteride vs. placebo in 18,882 low to average risk men aged 55 years or over [55]. After a 7-year follow up, the trial reported a 24.8% reduction in prostate cancer cases overall (803 vs. 1147, *P* < 0.0001), but high-grade tumours (Gleason grade ≥7) were more common in the treated group (280 vs. 337, *P* = 0.005). This has led to much debate, but after further follow up for a median of 18 years, no excess in prostate cancer deaths has been observed [56]. It has been suggested that the reason for the increased detection of high-grade tumours was due to a reduction in prostate size associated with finasteride treatment, which led to more accurate biopsies being taken. This in turn may have altered the assessment of Gleason grade, again reinforcing the need for long-term follow up to discover the full impact of a clinical intervention.

## Treatment trials

Long-term follow up often needs to be seen in a different context in treatment trials, as often the norm is for some form of treatment where there is considerable evidence for a short term benefit, and the ethics and safety of giving no or less treatment is a bigger concern. Thus, the main question is whether a long-term benefit exists when short-term efficacy is well established, and if it outweighs any late adverse effects.

An early example of the importance of long-term follow up in treatment trials comes from a UKCCR overview of the use of radiotherapy in early breast cancer. This overview of four early trials, initiated between 1949 and 1974, included 4148 deaths in 7842 women. No impact on deaths was seen in the first 10 years of follow up, but it was found that the regimens used at that time led to an increase in cardiac deaths for left-sided breast cancer after 10 years of follow up [57, 58]. As a result of these findings, newer radiotherapy regimens have been developed and an Early Breast Cancer Trials Coordinating Group (EBCTCG) overview has demonstrated that these regimens have led to reductions in recurrences and deaths from breast cancer, and with minimal impact on non-breast cancer deaths [59], and minimal cardiac toxicity [60, 61].

Another area where long-term follow up has provided important information has been for the use of adjuvant endocrine therapy in early breast cancer. Notable in this area are the overviews conducted for tamoxifen by the EBCTCG. Among other things, they showed that in 10,645 oestrogen receptor-positive tumours, 5 years of tamoxifen led to a 50% reduction in recurrence in the first 5 years of follow up, a further 30% reduction in years 5–9, but no further effect in years 10–14 [62]. This is in contrast to the prevention trials, where 5 years of tamoxifen prevented new breast cancers for at least 20 years [33]. For breast cancer mortality, a one-third reduction was seen in the overview, a reduction sustained for at least 15 years. In contrast to the recurrence data, mortality reductions were as strong in years 10–14 after treatment as in earlier years (Relative Risk = 0.71, 0.66 and 0.68 in each 5-year period). Tamoxifen had little effect on deaths from other causes, so that there was a substantial effect on overall mortality. It was also shown that tamoxifen had little effect on breast cancer recurrence or mortality for the 5984 oestrogen receptor poor/negative breast cancers [62].

Several large trials of aromatase inhibitors vs. tamoxifen for 5 years as adjuvant therapy in postmenopausal women have been conducted. In addition to straight two-arm comparisons, some have also compared trials which switch between aromatase inhibitors and tamoxifen after 2–3 years of initial treatment [63]. This analysis showed a larger effect for an aromatase inhibitor during its use, but not afterwards. A mortality benefit was also seen. Only one trial (ATAC) has reported even moderately long-term follow up, which has now been extended to a median of 10 years [64]. Serious side-effects were lower with anastrozole than tamoxifen.

Improvements in progression rates do not always translate into disease mortality reductions. One trial of radiotherapy for prostate cancer [65] reported improvements in biochemical progression-free survival with dose escalated radiotherapy vs. a conventional dose, which have been maintained for a median of 10 years, but this has not translated into improvements in overall survival. An overview of three available subsequent radiotherapy trials with a median follow up between 60 and 78 months was unable to establish a difference in event free survival between upfront or early salvage radiotherapy, and noted that the latter produced fewer side-effects [66]. Longer follow up, or a larger trial may be needed to see if there is an effect.

Longer term toxicity issues can also negate any improvements seen in progression markers. Mauch et al. [67] noted the excess mortality from cardiovascular disease compared with the general population in both arms of a trial of radiotherapy vs. radiotherapy and chemotherapy in Hodgkin’s disease. In an overview of 8 trials of more vs. less radiotherapy in 1974 patients, and 13 trials in 1688 patients of adding chemotherapy to radiotherapy, Specht et al. [68] reported a one-third reduction in recurrence with added radiotherapy, and a halving with added chemotherapy. However, after 10 years of follow up, no significant effect was seen on mortality, suggesting that less intensive primary treatment—particularly a reduction in radiotherapy field—may have achieved the same results. Endpoints that are surrogates for disease-specific survival, such as recurrence, metastatic spread, or increases in tumour size or grade can be indicators of better survival, but as noted above, these do not always translate into reduced disease-specific mortality [69].

Because of the longer number of years at risk after cancer treatment, long-term follow up is especially important for childhood and adolescent cancers. A range of different issues need to be addressed, including effects on mental development and childbearing. Partly because of the heterogeneity of these cancers, this is rarely done within a clinical trial, but efforts to establish registries to create large cohorts to document late side-effects are now being made [70–72]. This is only a start, and more resources and effort are needed to develop this area.

Not all treatment questions can be answered by a single long-term follow up analysis. Factors other than follow up duration include sample size, choice of study endpoints, aggressiveness of applying salvage therapies, and subgroups with differing prognoses [73]. Adequate sample size is essential and can be determined by standard power calculations. A common question is whether reducing disease recurrence or progression translates into an improvement in survival. Often, overall survival is used for the latter. While this clearly has value as a bottom line, for diseases in which only a small to moderate number of patients actually die from the disease under study, such as early breast or prostate cancer, a substantial loss of power can occur due to deaths from other causes for which no treatment effect is anticipated and deaths from the disease under study can be a more powerful and useful measure of efficacy [74]. Differences in other causes of death that might be due to treatment are important, but are usually specific to a particular site, and often are not captured in an ‘all other cause’ mortality assessment.

Another issue is comparing up-front treatment with salvage therapy, often studied for the use of radiotherapy. Here the threshold for using salvage therapy can be important. For example, in intermediate-risk or high-risk, localised or locally advanced prostate cancer, Vale et al. [66] found salvage therapy to be equally effective, and that it reduced the number of men with size effects.

In addition, treatments may be more or less effective in different subgroups. It is clearly clinically important to identify any heterogeneity in response, but the reporting of apparent subgroup differences is one of the most common errors in analysing clinical trial results [75, 76]. Often numerous subgroups are examined, and the chance that one is truly significantly different at, say, a 5% level, is much less than the 95% suggested by this nominal two-sided *P*-value, due to the multiple comparisons being made. A Bonferroni or similar correction should be made. This requires specifying the number of subgroups to be investigated, and often can be problematic. Also any difference should be based on a significant interaction between a subgroup and the remaining trial population, rather than simply a significant effect in a subgroup [77].

In other reports, randomised studies are mixed with observational studies to increase sample size, but the potential for biased allocation in the observational studies still exists [73], although it may be less than for short term follow up.

## Summary

Clinically important findings can arise several years after treatment is completed, and often after formal follow up is stopped. An early analysis can give a distorted view of a treatment’s value. Especially in the prevention setting, unfavourable side-effects often occur early, and well before any benefits become apparent. In addition, favourable early effects on, e.g., recurrence, may or may not be maintained in the longer term, and may or may not lead to reductions in disease-specific mortality. Late treatment-related side-effects can also be uncovered, which can be particularly important when the prognosis is good. Many countries have national databases that can usually provide cause specific mortality, although recurrence and side effect data are less common. Linking basic long-term data to earlier clinical records can lead to valuable additional findings for a randomised clinical trial, and can provide important new findings that are more reliable than non-randomised comparisons.

## Data Availability

No new data was created or analysed in this report
